# How Interpersonal Distance Between Avatar and Human Influences Facial Affect Recognition in Immersive Virtual Reality

**DOI:** 10.3389/fpsyg.2021.675515

**Published:** 2021-07-15

**Authors:** Juan del Aguila, Luz M. González-Gualda, María Angeles Játiva, Patricia Fernández-Sotos, Antonio Fernández-Caballero, Arturo S. García

**Affiliations:** ^1^Complejo Hospitalario Universitario de Albacete (CHUA), Servicio de Salud de Castilla-La Mancha, Albacete, Spain; ^2^Instituto de Investigación en Informática de Albacete, Universidad de Castilla-La Mancha, Albacete, Spain; ^3^CIBERSAM (Biomedical Research Networking Centre in Mental Health), Madrid, Spain; ^4^Departamento de Sistemas Informáticos, Universidad de Castilla-La Mancha, Albacete, Spain

**Keywords:** virtual reality, facial affect recognition, interpersonal distance, emotion identification, reaction time

## Abstract

**Purpose:** The purpose of this study was to determine the optimal interpersonal distance (IPD) between humans and affective avatars in facial affect recognition in immersive virtual reality (IVR). The ideal IPD is the one in which the humans show the highest number of hits and the shortest reaction times in recognizing the emotions displayed by avatars. The results should help design future therapies to remedy facial affect recognition deficits.

**Methods:** A group of 39 healthy volunteers participated in an experiment in which participants were shown 65 dynamic faces in IVR and had to identify six basic emotions plus neutral expression presented by the avatars. We decided to limit the experiment to five different distances: D1 (35 cm), D2 (55 cm), D3 (75 cm), D4 (95 cm), and D5 (115 cm), all belonging to the intimate and personal interpersonal spaces. Of the total of 65 faces, 13 faces were presented for each of the included distances. The views were shown at different angles: 50% in frontal view, 25% from the right profile, and 25% from the left profile. The order of appearance of the faces presented to each participant was randomized.

**Results:** The overall success rate in facial emotion identification was 90.33%, being D3 the IPD with the best overall emotional recognition hits, although statistically significant differences could not be found between the IPDs. Consistent with results obtained in previous studies, identification rates for negative emotions were higher with increasing IPD, whereas the recognition task improved for positive emotions when IPD was closer. In addition, the study revealed irregular behavior in the facial detection of the emotion surprise.

**Conclusions:** IVR allows us to reliably assess facial emotion recognition using dynamic avatars as all the IPDs tested showed to be effective. However, no statistically significant differences in facial emotion recognition were found among the different IPDs.

## 1. Introduction

Virtual reality (VR) is a powerful tool that allows the creation of lifelike environments and situations by imitating the physical world digitally. The technology has increasingly gained impulse since the 1990s (Rothbaum, 2009; Fernández-Sotos et al., 2019). At the beginning, most of the research focused on the processes underlying anxiety disorders and their treatment. Thus, VR established itself as a means of investigating threat perception, fear, and exposure processing (Glotzbach-Schoon et al., 2013; Shiban et al., 2013; Diemer et al., 2015). In recent years, research has been extended to other mental disorders such as major depressive disorder, bipolar disorder, and schizophrenia (Chen et al., 2021; Vass et al., 2021). This body of research has concluded that VR is a valuable tool for assessing the presence of symptoms in valid and controlled settings (Rus-Calafell et al., 2018; Browning et al., 2020; Fernández-Sotos et al., 2020; Hørlyck et al., 2021). It has the potential to facilitate the learning of new emotional and behavioral responses and can be applied to cognitive rehabilitation (Burin et al., 2020) and social cognition training interventions (Rus-Calafell et al., 2018).

VR, and especially immersive VR (IVR), offers researchers unprecedented opportunities to investigate human response (Snoswell and Snoswell, 2019). More than a single technology, IVR is a growing set of tools and techniques that create the psychological sensation of being in an alternative space, allowing physical immersion in a 3D environment and interaction with the virtual world as part of vivid and realistic experiences (Slater, 2009; Diemer et al., 2015). This has proved particularly attractive for research into pathological processes in a number of mental disorders (Tambone et al., 2021). More concretely, there is consistent evidence that patients with various neuropsychiatric disorders experience significant difficulty in accurately recognizing emotions expressed by others (Penton-Voak et al., 2017). As opposed to traditional stimuli based on static pictures, VR may use controlled dynamic avatars to represent different emotional states and interact with the participant (Gutiérrez-Maldonado et al., 2014; Fernández-Caballero et al., 2017). In this respect, dynamic facial expressions rendered by virtual humans (avatars) may generate an intense emotional experience in the participant and facilitate successful emotional recognition (Sato and Yoshikawa, 2007). Furthermore, avatars can be modeled with any combination of race, age and gender, observed from any angle, under any lighting conditions and in any social context (Banakou et al., 2020). All this allows simulating social interactions similar to reality, providing therapists with the possibility to control and manipulate the behavior of avatars for assessing and training affect recognition skills.

Very recently, our research team has carried out the validation of a set of dynamic avatars for the task of facial affect recognition (Fernández-Sotos et al., 2021). The validation was performed on a healthy population with the intention of being able to make a comparison in the near future with patients. However, the facial emotions of the avatars were always presented to the healthy participants at an equal distance. Therefore, it seemed important to us, with a view to future therapies for remediation of facial affect recognition deficits (Monferrer et al., 2021; Muros et al., 2021), to determine the optimal interpersonal distance (IPD) in healthy people at recognizing these emotions on affective avatars in IVR.

IPD, defined as the distance an individual maintains from another to avoid intrusion into personal space, is an intrinsic component of interaction with others and is of particular value in social processes (Hayduk, 1978; Iachini et al., 2015). A very recent work has proposed a theoretical framework linking peripersonal action and interpersonal social spaces that sheds light on social behaviors in populations with socioemotional deficits (Coello and Cartaud, 2021). Previous studies have shown that people tend to move away from interlocutors when they feel hostile and uncomfortable and to reduce IPD when they feel friendly, comfortable or intimate (Lloyd, 2009). Facial expression of a threatening emotion such as anger will increase IPD, whereas IPD will be less in the presence of a friendly or familiar face (Hall, 1963; Marsh et al., 2005; Welsch et al., 2020). In addition, studies have shown interaction between facial expression, physiological response, and IPD (e.g., Cartaud et al., 2018). IPD is a fundamental factor of proper facial emotion recognition and is influenced by different variables such as social norms and participant characteristics, in terms of gender (Ozdemir, 2008; Iachini et al., 2016), age (Iachini et al., 2016; Pochwatko et al., 2021), and psychopathology (Holt et al., 2015; Asada et al., 2016; Nandrino et al., 2017). However, the studies that have investigated the “ideal” or preferred IPD between a participant and avatars in IVR are scarce and cutting-edge (Ruggiero et al., 2021).

In this work, the ideal IPD is considered as the one where the participant shows a higher number of hits and shorter reaction times in the recognition of the emotions shown. The results of this study would help to employ a single IPD to assess differences in facial affect recognition between healthy individuals and patients. For this purpose, five different IPDs have been included in the experiment. The same tool previously designed by the research team (García et al., 2020b) to validate a set of avatar faces on a healthy population was used to carry out the emotion recognition task.

## 2. Materials and Methods

### 2.1. Participants

A group of 39 healthy volunteers (20 women and 19 men) took part in this experiment. They were recruited in Albacete (Spain) during a 3-month cross-sectional study (from September to December 2020). The inclusion criteria were being aged between 20 and 79 years, having no previous diagnosis of mental illness, no personal history of medical illness, and no first-degree family history of psychosis. The mean age of the group was *M* = 42.15, *SD* = 13.54, the maximum was 71 and the minimum 25. None of them took part in previous experiments conducted by our research group. The study was conducted according to the guidelines of the Declaration of Helsinki, and approved by the Clinical Research Ethics Committee of the Complejo Hospitalario Universitario de Albacete (protocol code 2019/07/073 and date of approval 24 September 2019). Informed consent was obtained from all subjects involved in the study.

To ensure that the sample size provided an adequate level of statistical power, a sensitivity test was performed using the G^*^Power program (version 3.1.9.7) to calculate the minimum required effect size. A critical value *d* = 0.536 was obtained for α = 0.05, power = 0.95 (1−β), one sample group with *n* = 39, a non-centrality parameter δ = 3.350 and a critical value *t* = 1.686. With respect to the effect size, what we have put at reference level is the critical value of Cohen's *d*, which is indeed medium.

### 2.2. Experimental Procedure

The Spanish version of the Positive and Negative Affect Scale (PANAS) (Watson et al., 1988) was administered. PANAS is a 20-item self-administered questionnaire measuring mood. The scale was administered in order to exclude participants with non-specific depressive symptoms. If a participant had a positive affect (PA) score of <25 (PA < 25) or a negative affect (NA) score of more than 35 (NA >35), he/she was excluded from the study.

Data collection took place in a single 45-min individual session, during which the participant was accompanied by a member of the research team at all times. The possibility of leaving the study after the start was offered upon the participant's request. This did not happen for any participant. At the beginning of the test, participants could practice for a few minutes to get accustomed to the use of the devices. No data were measured during practice.

**Figure 1:**
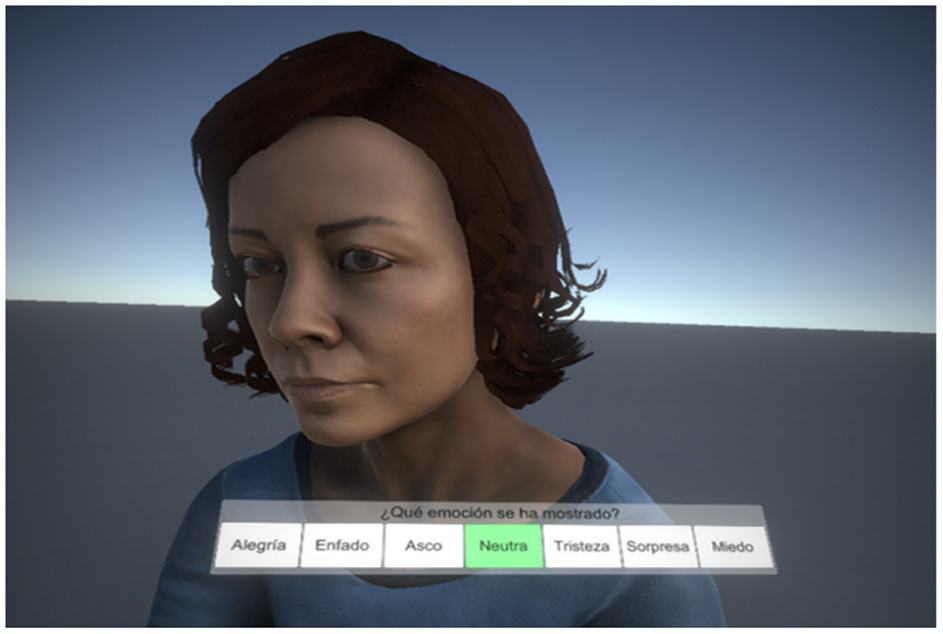
Panel used by the participants to select the emotion depicted by the virtual human.

During the experiment, all participants were shown 65 dynamic faces in IVR on which they had to identify the basic emotions presented, always starting from and ending at the *Neutral* expression, with a total presentation speed of 2 s. The basic emotions shown were *Joy, Sadness, Anger, Fear, Disgust*, and *Surprise*. Right after the emotion was blended to the *Neutral* expression, a panel with seven possible alternatives (the six basic emotions plus the *Neutral* expression) appeared under the avatar's head (see Figure 1). This allowed the participants to select the correct emotion. It is worth noting that even though the panel always appeared under the avatars' head, its size changed depending on the distance to the participant so that it had the same relative size regardless of the observers' point of view. Upon the participant selected an option from the panel, the whole environment was faded-out to a light-gray background in a transition that lasted 2.5 s, and a new character was faded-in. The response selected by the user was recorded along with the response time, even though the participants were not instructed to respond as quickly as possible.

Facial dynamism refers to the movement of the parts of the face involved to express an emotion and to a slight emotion-specific movement of the head and neck. For example, *Fear* involves moving the neck joint backwards, while *Anger* moves the avatar's head forward. This level of dynamism was selected because better results were obtained in a previous experiment (García et al., 2020b), in which the design of the emotions was described in depth. Participants had to identify the emotion expressed on the virtual faces from the seven options offered (including the *Neutral* expression). Of the total of 65 faces, each basic emotion was presented 10 times (2 times per each IPD, with two levels of intensity) and the *Neutral* expression was presented 5 times (1 time per IPD), making a total amount of 13 faces with each of the IPDs included. Different angles were shown: 50% from frontal view, 25% in right profile, and 25% in left profile. The order of appearance of the faces presented to each participant was randomized, as well as the avatars' gender (50% male and 50% female, for each participant). Therefore, the presentation order of the emotions, the virtual characters depicting them, the IPDs, and the camera angles were different from one user to another.

### 2.3. Experimental Setup

The IPD is known to vary with social function, giving rise to different zones: intimate space (defined as 0–45 cm) reserved for close family members, children and pets; personal space (46–122 cm) used in conversations with friends and group discussions; social space (123–365 cm) reserved for strangers, newly formed groups and new acquaintances; and public space (distance >365 cm), used in speeches, lectures, and theater (Hall, 1966). We decided to limit the experiment to five different IPDs: D1 (35 cm), D2 (55 cm), D3 (75 cm), D4 (95 cm), and D5 (115 cm), all of them belonging to the intimate and personal spaces, which are the spaces where the easiest future learning of emotions is foreseen in IVR setups.

We used an immersive version of the software originally designed for facial affect recognition under non-immersive virtual reality (García et al., 2020b; Fernández-Sotos et al., 2021). Therefore, the avatars used and the expressions they depict have already been validated by a sample of 204 healthy volunteers (50% women and 50% men) using a non-immersive display. The sample was stratified in 3 age ranges and education levels considering the level of education of the Spanish population in 2017. It is worth noting that the emotions were designed following the well-known Facial Action Coding System (FACS) (Ekman and Friesen, 1978).

**Figure 2:**
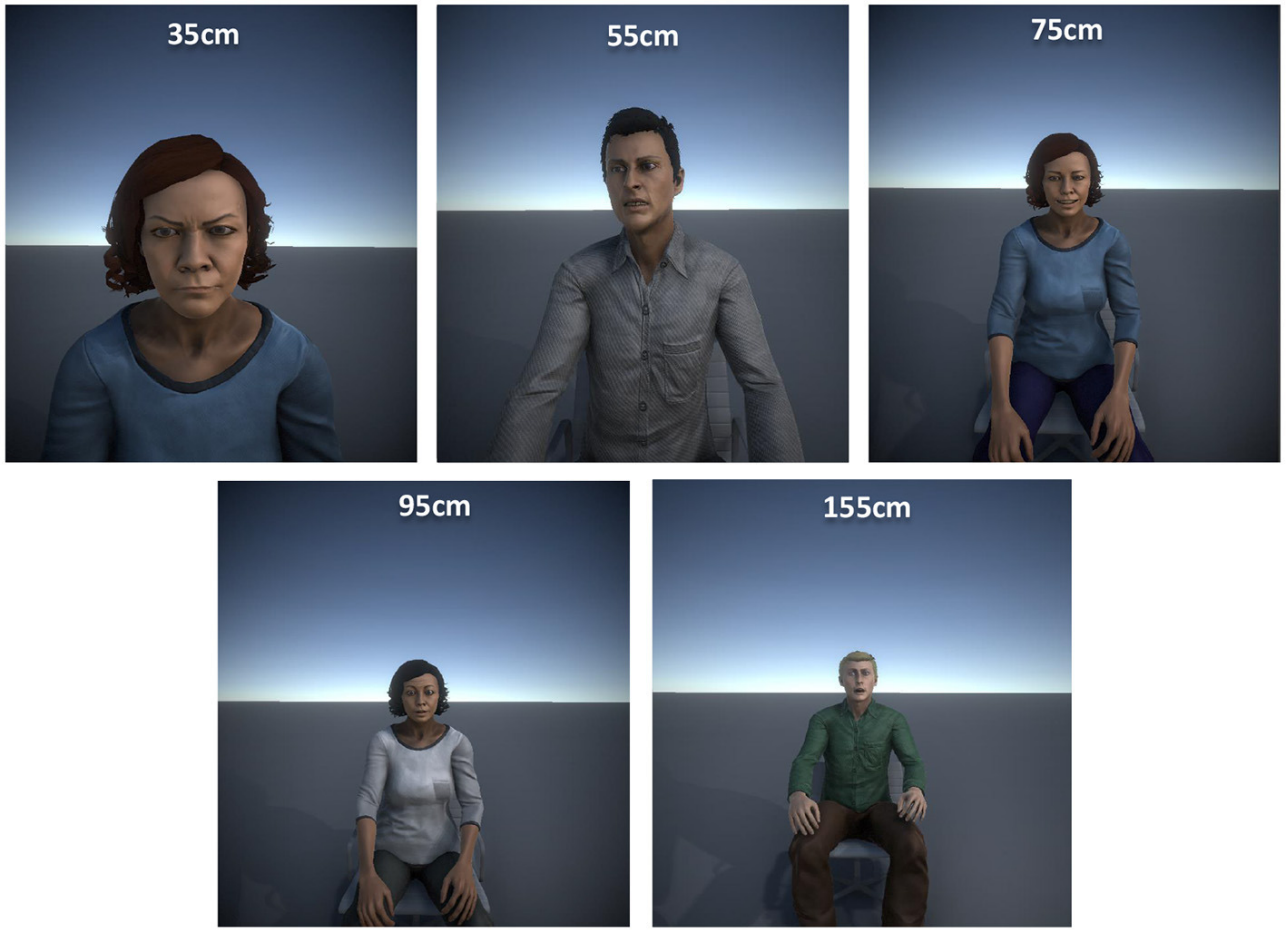
Virtual humans as seen by participants using the HMD for each of the distances considered.

The experiment was designed as a seated IVR experience. Participants wearing a head mounted display (HMD) remained seated in front of an avatar that was also seated. The participants did not see their own body nor a virtual representation during the tests, so they were instructed to focus only on the avatar in front of them. Figure 2 provides examples of the viewpoint of the participants for the five different IPDs considered. Since they were immersed in the virtual environment, a gamepad controller was used to select one of the seven alternatives displayed in the response panel (Figure 1). The selection was made using the directional pad or any of the sticks, while confirmation was made by pressing any of the buttons. As mentioned in section 2.2, the participants had some time to practice and get used to the devices and the button configuration.

Therefore, the materials used to carry out the experiment consisted of a laptop computer provided with a 17.3” screen, an Intel Core i7-9750H, 16 GB of RAM and an NVIDIA RTX2070 graphics card; a standard gamepad and a FOVE HMD with an OLED display of 2,560 × 1,440 pixels and a field of view of up to 100 degrees. A preliminary study was conducted to identify the range of distances achievable with this hardware configuration. It was experimentally identified that 115 cm was the maximum distance at which emotions could be identified considering the number of pixels available for the face in the virtual characters. Beyond that distance, the resolution of the face was considered too small for identification.

### 2.4. Data Analysis

The responses of the users to each face presented along with the number of correct emotion identifications and response times were stored in a CSV file. Microsoft Excel and IMB SPSS Statistics v24 were used to analyze this data. Descriptive statistics were used to describe the sample data, while hypothesis testing techniques were used to test whether there was a significant difference between the distance of presentation of the emotion. The data for the number of correct answers and for reaction time did not follow a normal distribution (Shapiro-Wilk *Z* = 0.568, *p* < 0.000 and *Z* = 0.796, *p* < 0.000, respectively). Therefore, we used non-parametric tests to compare the results for each distance (Friedman and Wilcoxon Signed Rank tests). The level of significance for the tests was established to 95%.

## 3. Results

Given that the main aim of this experiment was on the impact of the presentation distance of the stimuli on emotion identification and reaction time, we will focus on this during the reporting of the results. Nonetheless, some general results are also discussed, namely the average emotion identification rates and the reaction times per emotion. The influence of the camera angle is also considered.

### 3.1. Emotion Identification Rates

The success rate in emotion identification was 90.33%, while the result per each emotion is displayed on Table 1. The rows represent the emotion presented to the participant, and the columns represent the emotion they believed the avatar was showing. As can be seen in the table, the results are above 86% for all the emotions but *Fear*. While it is still high (73.6%), 22.3% of the participants made a mistake and selected *Surprise* instead (row *Fear*, column *Surprise*). Apart from that, it is also worth noting that 7.7% selected *Anger* when *Disgust* was presented to them (row *Disgust*, column *Anger*).

**Table 1:** Emotion recognition rate for each emotion depicted. The average rate is 90.3%.

	**Neutral**	**Surprise**	**Fear**	**Anger**	**Disgust**	**Joy**	**Sadness**
Neutral	**96.9%**	0.0%	0.0%	0.5%	1.0%	0.0%	1.5%
Surprise	1.0%	**92.8%**	4.9%	0.0%	0.5%	0.0%	0.8%
Fear	2.3%	22.3%	**73.6%**	0.0%	0.8%	0.0%	1.0%
Anger	1.3%	0.5%	0.5%	**96.4%**	0.5%	0.3%	0.5%
Disgust	2.3%	0.3%	0.3%	7.7%	**89.0%**	0.3%	0.3%
Joy	1.5%	1.5%	0.0%	0.3%	0.0%	**96.7%**	0.0%
Sadness	4.4%	1.5%	1.8%	3.6%	1.8%	0.0%	**86.9%**

*Columns, emotions recognized; Rows, emotions presented. Bold stands for the diagonal of the correlation matrix*.

### 3.2. Influence of Interpersonal Distance in Emotion Identification

**Figure 3:**
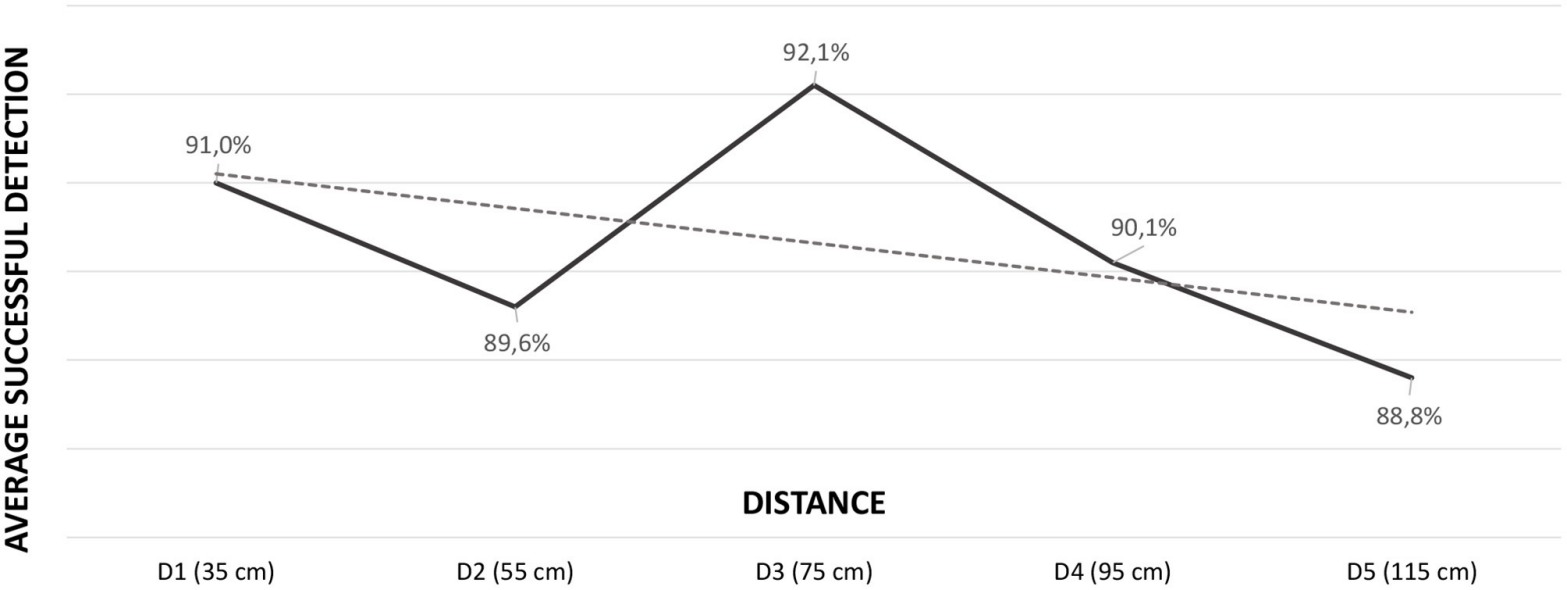
Average successful recognition rate per distance, including a trend dashed line with a slope of −0.0038.

Table 2 presents the results obtained in the emotion identification for each one of the IPDs selected. The average recognition rates per IPD are also included in the table. The results do not differ much from the ones presented in Table 1, but a slight reduction in the average rates is apparent as the IPD increases, as can also be observed in the gray line of Figure 3, which refers to the overall emotion identification results (the trend line is depicted as a dashed line and has a small negative slope of −0.0038%). We used the Friedman test to try to find statistically significant differences in the total number of correct answers per distance. However, the application of the test could not reveal significant differences in the data [$\chi_{\left(4\right)}^{2}=4.423,p=0.352$].

**Table 2:** Emotion recognition rates and average values for each interpersonal distance.

**D1 (35 cm). Avg. 91.03%**								**D2 (55 cm). Avg. 89.56%**						
	**Neutral**	**Surprise**	**Fear**	**Anger**	**Disgust**	**Joy**	**Sadness**	**Neutral**	**Surprise**	**Fear**	**Anger**	**Disgust**	**Joy**	**Sadness**
Neutral	**97.4%**	0.0%	0.0%	0.0%	0.0%	0.0%	2.6%	**97.4%**	0.0%	0.0%	0.0%	0.0%	0.0%	2.6%
Surprise	1.3%	**96.2%**	1.3%	0.0%	0.0%	0.0%	1.3%	2.6%	**89.7%**	6.4%	0.0%	0.0%	0.0%	1.3%
Fear	3.8%	23.1%	**73.1%**	0.0%	0.0%	0.0%	0.0%	2.6%	19.2%	**76.9%**	0.0%	0.0%	0.0%	1.3%
Anger	1.3%	1.3%	0.0%	**96.2%**	0.0%	1.3%	0.0%	1.3%	1.3%	0.0%	**96.2%**	0.0%	0.0%	1.3%
Disgust	1.3%	0.0%	0.0%	6.4%	**91.0%**	0.0%	1.3%	3.8%	1.3%	0.0%	10.3%	**84.6%**	0.0%	0.0%
Joy	0.0%	1.3%	0.0%	0.0%	0.0%	**98.7%**	0.0%	1.3%	1.3%	0.0%	0.0%	0.0%	**97.4%**	0.0%
Sadness	5.1%	1.3%	2.6%	5.1%	1.3%	0.0%	**84.6%**	6.4%	3.8%	1.3%	3.8%	0.0%	0.0%	**84.6%**
**D3 (75 cm). Avg. 92.12%**								**D4 (95 cm). Avg. 90.11%**						
	Neutral	Surprise	Fear	Anger	Disgust	Joy	Sadness	Neutral	Surprise	Fear	Anger	Disgust	Joy	Sadness
Neutral	**97.4%**	0.0%	0.0%	0.0%	2.6%	0.0%	0.0%	**97.4%**	0.0%	0.0%	0.0%	0.0%	0.0%	2.6%
Surprise	0.0%	**94.9%**	3.8%	0.0%	1.3%	0.0%	0.0%	1.3%	**89.7%**	6.4%	0.0%	1.3%	0.0%	1.3%
Fear	1.3%	25.6%	**73.1%**	0.0%	0.0%	0.0%	0.0%	2.6%	23.1%	**73.1%**	0.0%	1.3%	0.0%	0.0%
Anger	0.0%	0.0%	1.3%	**98.7%**	0.0%	0.0%	0.0%	2.6%	0.0%	0.0%	**96.2%**	0.0%	0.0%	1.3%
Disgust	1.3%	0.0%	0.0%	7.7%	**89.7%**	1.3%	0.0%	2.6%	0.0%	1.3%	7.7%	**88.5%**	0.0%	0.0%
Joy	1.3%	1.3%	0.0%	0.0%	0.0%	**97.4%**	0.0%	2.6%	1.3%	0.0%	1.3%	0.0%	**94.9%**	0.0%
Sadness	2.6%	0.0%	1.3%	2.6%	0.0%	0.0%	**93.6%**	2.6%	0.0%	2.6%	1.3%	2.6%	0.0%	**91.0%**
**D5 (115 cm). Avg. 88.83%**														
	Neutral	Surprise	Fear	Anger	Disgust	Joy	Sadness							
Neutral	**94.9%**	0.0%	0.0%	2.6%	2.6%	0.0%	0.0%							
Surprise	0.0%	**93.6%**	6.4%	0.0%	0.0%	0.0%	0.0%							
Fear	1.3%	20.5%	**71.8%**	0.0%	2.6%	0.0%	3.8%							
Anger	1.3%	0.0%	1.3%	**94.9%**	2.6%	0.0%	0.0%							
Disgust	2.6%	0.0%	0.0%	6.4%	**91.0%**	0.0%	0.0%							
Joy	2.6%	2.6%	0.0%	0.0%	0.0%	**94.9%**	0.0%							
Sadness	5.1%	2.6%	1.3%	5.1%	5.1%	0.0%	**80.8%**							

*Bold stands for the diagonal of the correlation matrix*.

**Figure 4:**
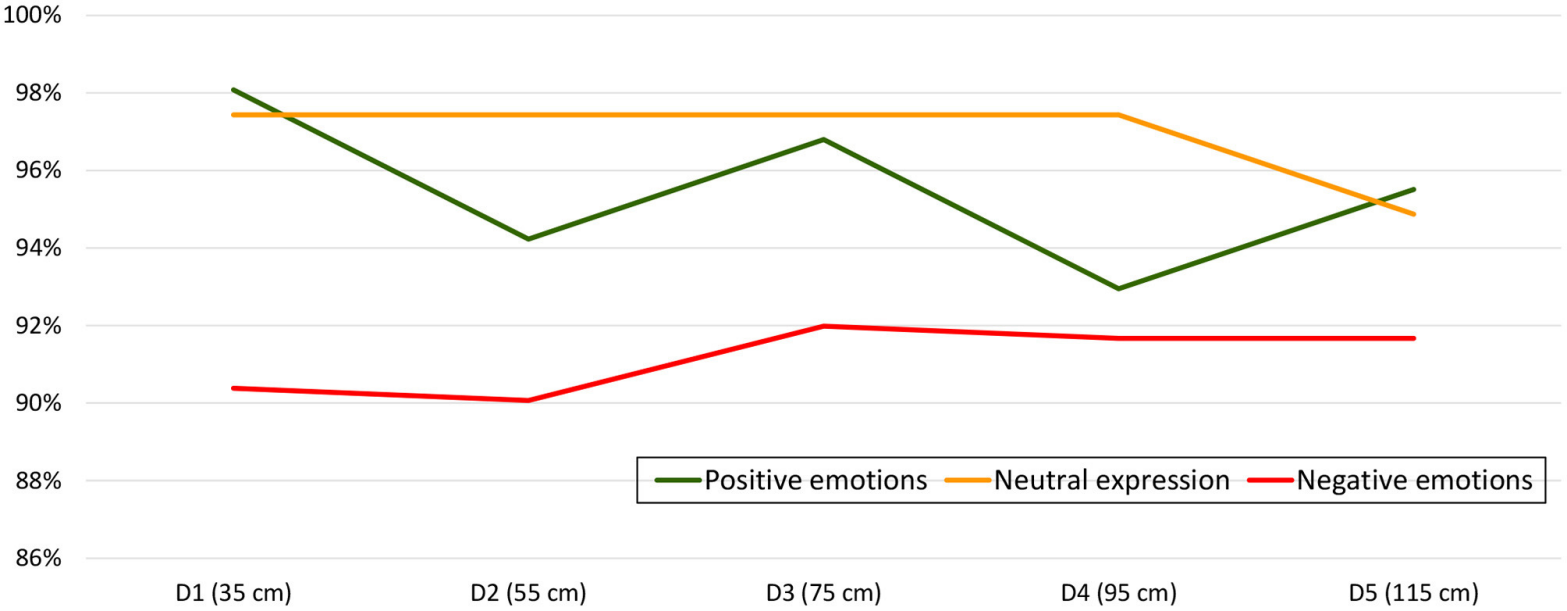
Average successful recognition rate per distance and affect category (positive, neutral, and negative emotions).

**Figure 5:**
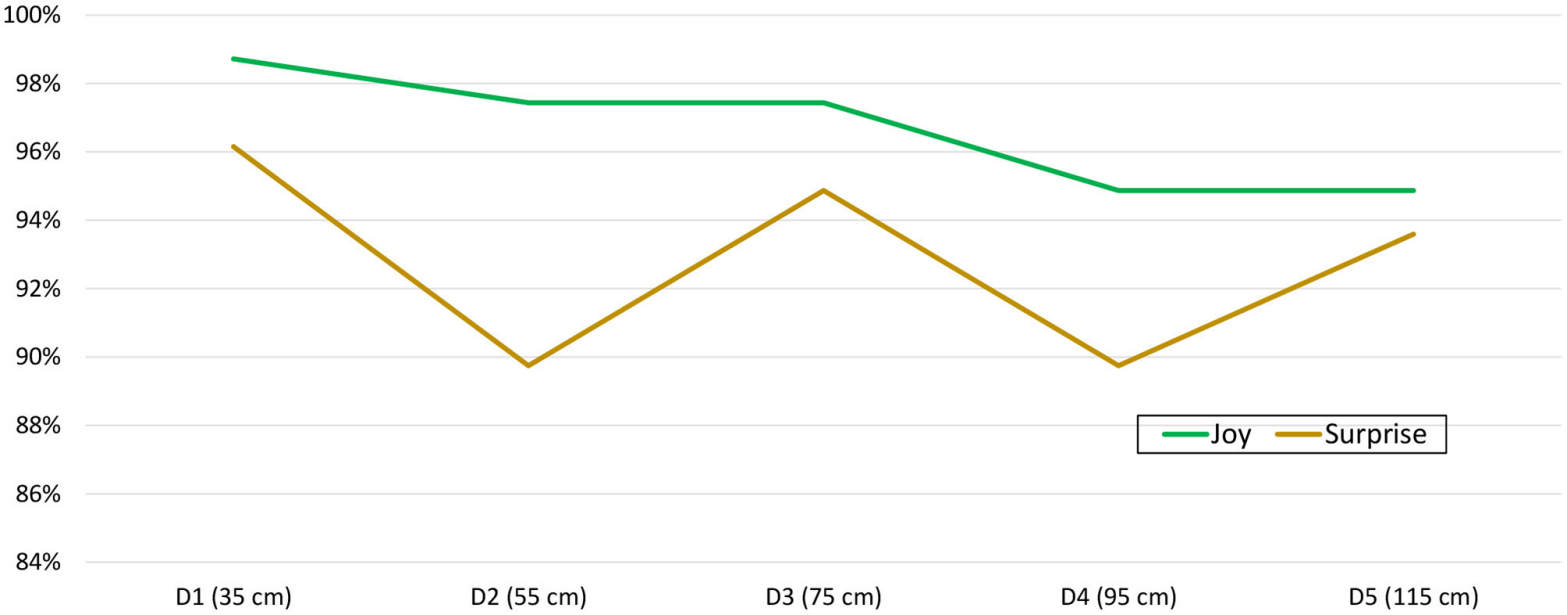
Average successful recognition rate per distance for the positive affect category (Joy and Surprise).

Although a trend toward a lower emotion recognition rate could be observed as the distance from the avatar to the human increased (see Figure 3), the behavior of the graph, especially for distances D2 and D3, was difficult to explain. Therefore, we also studied each emotion separately in order to find out if any of them was more liable to the effect of IPD. This study per emotion and IPD did not reveal statistically significant differences either, as can be seen in Table 3. Therefore, the relationship between distance and emotion recognition ratio was studied, now classifying emotions into positive, neutral and negative affective categories. It was concluded that positive emotions were responsible for the strange effect detected at intermediate IPDs (see Figure 4). Again, no statistically significant differences were found in the positive, negative and neutral affect categories per distance [$\chi_{\left(4\right)}^{2}=5.352,p=0.253$, $\chi_{\left(4\right)}^{2}=0.895,p=0.925$ and $\chi_{\left(4\right)}^{2}=1.000,p=0.910$, respectively]. Lastly, *Joy* and *Surprise* were studied separately. The results were drawn in Figure 5. Thus, it became apparent that it was the *Surprise* emotion that had caused the irregular shape of the curve of average recognition rates by IPD.

**Table 3:** Results of the application of the Friedman Test [$\chi_{\left(4\right)}^{2}$] to find differences in emotion identification per IPD on each individual emotion.

**Emotion**	**chi-square**	***p*-value**
Neutral	$\chi_{\left(4\right)}^{2}=1.000$	*p* = 0.910
Surprise	$\chi_{\left(4\right)}^{2}=3.553$	*p* = 0.470
Fear	$\chi_{\left(4\right)}^{2}=1.784$	*p* = 0.775
Anger	$\chi_{\left(4\right)}^{2}=3.429$	*p* = 0.489
Disgust	$\chi_{\left(4\right)}^{2}=2.909$	*p* = 0.573
Joy	$\chi_{\left(4\right)}^{2}=8.000$	*p* = 0.092
Sadness	$\chi_{\left(4\right)}^{2}=9.161$	*p* = 0.057
All	$\chi_{\left(4\right)}^{2}=4.423$	*p* = 0.352

*No statistically significant differences were found*.

### 3.3. Influence of Distance and Camera Angles in the Emotion Identification

Tables 4, 5 present the emotion recognition rates per each one of the distances for the different emotion presentation angles (cameras) used in the experiment, namely *front* and *side*, respectively. These results show a slight decline in the emotion identification rates for *side* views. In order to test if the observed differences are significant, we firstly compared the results on emotion identification for the two cameras. The aim of this comparison was to get insight into the impact of camera angle in emotion identification in order to test if this impact is similar on each of the distances. The Wilcoxon Signed Ranks Test was not able to find significant differences in the data obtained for each camera angle (*Z* = −1.160, *p* = 0.246). Similar results are obtained for each emotion.

**Table 4:** Emotion recognition rates and average values of the FRONT view for each interpersonal distance.

**D1 (35 cm). Avg. 94.83%**								**D2 (55 cm). Avg. 90.79%**						
	**Neutral**	**Surprise**	**Fear**	**Anger**	**Disgust**	**Joy**	**Sadness**	**Neutral**	**Surprise**	**Fear**	**Anger**	**Disgust**	**Joy**	**Sadness**
Neutral	**94.4%**	0.0%	0.0%	0.0%	0.0%	0.0%	5.6%	**100.0%**	0.0%	0.0%	0.0%	0.0%	0.0%	0.0%
Surprise	3.0%	**93.9%**	3.0%	0.0%	0.0%	0.0%	0.0%	0.0%	**86.5%**	10.8%	0.0%	0.0%	0.0%	2.7%
Fear	0.0%	13.2%	**86.8%**	0.0%	0.0%	0.0%	0.0%	2.7%	13.5%	**81.1%**	0.0%	0.0%	0.0%	2.7%
Anger	2.9%	0.0%	0.0%	**97.1%**	0.0%	0.0%	0.0%	2.4%	0.0%	0.0%	**95.1%**	0.0%	0.0%	2.4%
Disgust	2.2%	0.0%	0.0%	0.0%	**97.8%**	0.0%	0.0%	4.7%	0.0%	0.0%	9.3%	**86.0%**	0.0%	0.0%
Joy	0.0%	0.0%	0.0%	0.0%	0.0%	**100.0%**	0.0%	0.0%	2.7%	0.0%	0.0%	0.0%	**97.3%**	0.0%
Sadness	3.1%	0.0%	0.0%	0.0%	3.1%	0.0%	**93.8%**	7.9%	0.0%	2.6%	0.0%	0.0%	0.0%	**89.5%**
**D3 (75 cm). Avg. 95.56%**								**D4 (95 cm). Avg. 92.29%**						
	Neutral	Surprise	Fear	Anger	Disgust	Joy	Sadness	Neutral	Surprise	Fear	Anger	Disgust	Joy	Sadness
Neutral	**95.5%**	0.0%	0.0%	0.0%	4.5%	0.0%	0.0%	**100.0%**	0.0%	0.0%	0.0%	0.0%	0.0%	0.0%
Surprise	0.0%	**97.5%**	2.5%	0.0%	0.0%	0.0%	0.0%	2.6%	**92.1%**	2.6%	0.0%	0.0%	0.0%	2.6%
Fear	0.0%	9.5%	**90.5%**	0.0%	0.0%	0.0%	0.0%	3.1%	15.6%	**78.1%**	0.0%	3.1%	0.0%	0.0%
Anger	0.0%	0.0%	0.0%	**100.0%**	0.0%	0.0%	0.0%	2.4%	0.0%	0.0%	**95.2%**	0.0%	0.0%	2.4%
Disgust	2.7%	0.0%	0.0%	2.7%	**94.6%**	0.0%	0.0%	5.6%	0.0%	0.0%	0.0%	**94.4%**	0.0%	0.0%
Joy	0.0%	0.0%	0.0%	0.0%	0.0%	**100.0%**	0.0%	4.5%	2.3%	0.0%	2.3%	0.0%	**90.9%**	0.0%
Sadness	2.3%	0.0%	2.3%	4.5%	0.0%	0.0%	**90.9%**	2.4%	0.0%	2.4%	0.0%	0.0%	0.0%	**95.2%**
**D5 (115 cm). Avg. 88.73%**								**ALL. Avg. 92.45%**						
	Neutral	Surprise	Fear	Anger	Disgust	Joy	Sadness	Neutral	Surprise	Fear	Anger	Disgust	Joy	Sadness
Neutral	**100.0%**	0.0%	0.0%	0.0%	0.0%	0.0%	0.0%	**97.9%**	0.0%	0.0%	0.0%	1.0%	0.0%	1.0%
Surprise	0.0%	**94.4%**	5.6%	0.0%	0.0%	0.0%	0.0%	1.1%	**92.9%**	4.9%	0.0%	0.0%	0.0%	1.1%
Fear	0.0%	17.5%	**75.0%**	0.0%	2.5%	0.0%	5.0%	1.1%	13.8%	**82.5%**	0.0%	1.1%	0.0%	1.6%
Anger	2.3%	0.0%	2.3%	**90.7%**	4.7%	0.0%	0.0%	2.0%	0.0%	0.5%	**95.6%**	1.0%	0.0%	1.0%
Disgust	5.9%	0.0%	0.0%	2.9%	**91.2%**	0.0%	0.0%	4.1%	0.0%	0.0%	3.1%	**92.8%**	0.0%	0.0%
Joy	2.9%	5.7%	0.0%	0.0%	0.0%	**91.4%**	0.0%	1.6%	2.1%	0.0%	0.5%	0.0%	**95.7%**	0.0%
Sadness	5.4%	2.7%	0.0%	5.4%	8.1%	0.0%	**78.4%**	4.1%	0.5%	1.6%	2.1%	2.1%	0.0%	**89.6%**

*Bold stands for the diagonal of the correlation matrix*.

**Table 5:** Emotion recognition rates and average values of the SIDE views for each interpersonal distance.

**D1 (35 cm). Avg. 87.25%**								**D2 (55 cm). Avg. 88.46%**						
	**Neutral**	**Surprise**	**Fear**	**Anger**	**Disgust**	**Joy**	**Sadness**	**Neutral**	**Surprise**	**Fear**	**Anger**	**Disgust**	**Joy**	**Sadness**
Neutral	**100.0%**	0.0%	0.0%	0.0%	0.0%	0.0%	0.0%	**95.7%**	0.0%	0.0%	0.0%	0.0%	0.0%	4.3%
Surprise	0.0%	**97.8%**	0.0%	0.0%	0.0%	0.0%	2.2%	4.9%	**92.7%**	2.4%	0.0%	0.0%	0.0%	0.0%
Fear	7.5%	32.5%	**60.0%**	0.0%	0.0%	0.0%	0.0%	2.4%	24.4%	**73.2%**	0.0%	0.0%	0.0%	0.0%
Anger	0.0%	2.3%	0.0%	**95.5%**	0.0%	2.3%	0.0%	0.0%	2.7%	0.0%	**97.3%**	0.0%	0.0%	0.0%
Disgust	0.0%	0.0%	0.0%	15.2%	**81.8%**	0.0%	3.0%	2.9%	2.9%	0.0%	11.4%	**82.9%**	0.0%	0.0%
Joy	0.0%	2.6%	0.0%	0.0%	0.0%	**97.4%**	0.0%	2.4%	0.0%	0.0%	0.0%	0.0%	**97.6%**	0.0%
Sadness	6.5%	2.2%	4.3%	8.7%	0.0%	0.0%	**78.3%**	5.0%	7.5%	0.0%	7.5%	0.0%	0.0%	**80.0%**
**D3 (75 cm). Avg. 88.59%**								**D4 (95 cm). Avg. 88.35%**						
	Neutral	Surprise	Fear	Anger	Disgust	Joy	Sadness	Neutral	Surprise	Fear	Anger	Disgust	Joy	Sadness
Neutral	**100.0%**	0.0%	0.0%	0.0%	0.0%	0.0%	0.0%	**94.7%**	0.0%	0.0%	0.0%	0.0%	0.0%	5.3%
Surprise	0.0%	**92.1%**	5.3%	0.0%	2.6%	0.0%	0.0%	0.0%	**87.5%**	10.0%	0.0%	2.5%	0.0%	0.0%
Fear	2.8%	44.4%	**52.8%**	0.0%	0.0%	0.0%	0.0%	2.2%	28.3%	**69.6%**	0.0%	0.0%	0.0%	0.0%
Anger	0.0%	0.0%	2.9%	**97.1%**	0.0%	0.0%	0.0%	2.8%	0.0%	0.0%	**97.2%**	0.0%	0.0%	0.0%
Disgust	0.0%	0.0%	0.0%	12.2%	**85.4%**	2.4%	0.0%	0.0%	0.0%	2.4%	14.3%	**83.3%**	0.0%	0.0%
Joy	2.2%	2.2%	0.0%	0.0%	0.0%	**95.7%**	0.0%	0.0%	0.0%	0.0%	0.0%	0.0%	**100.0%**	0.0%
Sadness	2.9%	0.0%	0.0%	0.0%	0.0%	0.0%	**97.1%**	2.8%	0.0%	2.8%	2.8%	5.6%	0.0%	**86.1%**
**D5 (115 cm). Avg. 88.81%**								**ALL. Avg. 88.30%**						
	Neutral	Surprise	Fear	Anger	Disgust	Joy	Sadness	Neutral	Surprise	Fear	Anger	Disgust	Joy	Sadness
Neutral	**88.9%**	0.0%	0.0%	5.6%	5.6%	0.0%	0.0%	**95.9%**	0.0%	0.0%	1.0%	1.0%	0.0%	2.0%
Surprise	0.0%	**92.9%**	7.1%	0.0%	0.0%	0.0%	0.0%	1.0%	**92.7%**	4.9%	0.0%	1.0%	0.0%	0.5%
Fear	2.6%	23.7%	**68.4%**	0.0%	2.6%	0.0%	2.6%	3.5%	30.3%	**65.2%**	0.0%	0.5%	0.0%	0.5%
Anger	0.0%	0.0%	0.0%	**100.0%**	0.0%	0.0%	0.0%	0.5%	1.1%	0.5%	**97.3%**	0.0%	0.5%	0.0%
Disgust	0.0%	0.0%	0.0%	9.1%	**90.9%**	0.0%	0.0%	0.5%	0.5%	0.5%	12.3%	**85.1%**	0.5%	0.5%
Joy	2.3%	0.0%	0.0%	0.0%	0.0%	**97.7%**	0.0%	1.5%	1.0%	0.0%	0.0%	0.0%	**97.5%**	0.0%
Sadness	4.9%	2.4%	2.4%	4.9%	2.4%	0.0%	**82.9%**	4.6%	2.5%	2.0%	5.1%	1.5%	0.0%	**84.3%**

*Bold stands for the diagonal of the correlation matrix*.

Our next step was to study the influence of the distance on the emotion identification for each one of the camera angles. With this, we wanted to study if the stimuli presentation distance had a different impact when using different presentation angles. No significant differences were found in the total number of correct answers per distance when the *front* camera was used (Friedman Test $\chi_{\left(4\right)}^{2}=4.469,p=0.346$). Similar results were obtained per each emotion. The results for the tests in which the *side* cameras were used are similar for the total number of correct answers [$\chi_{\left(4\right)}^{2}=3.785,p=0.436$] and for individual emotions.

Finally, we looked for significant differences in the results obtained for each camera angle per each one of the IPDs. For D1, a significant difference was found for *Disgust* (*Z* = −2.043, *p* = 0.041), being the number of correct answers higher for the *front* camera. No significant differences were found for D2, contrarily to D3 in which *Fear* obtained a significantly higher number of correct answers for the *front* camera (*Z* = −2.567, *p* = 0.010). For the remaining distances (D4 and D5), the tests found no significant differences in the data. The results of all emotions have been included in Table 6.

**Table 6:** Results of the application of the Wilcoxon Signed Ranks Test (*Z*) to find differences in emotion identification between camera angles (front, side) per each IPD.

	**D1 (35 cm)**	**D2 (55 cm)**	**D3 (75 cm)**	**D4 (95 cm)**	**D5 (115 cm)**
Neutral	−0.649, *p* = 0.516	−0.973, *p* = 0.330	−0.649, *p* = 0.516	−0.324, *p* = 0.746	−0.822, *p* = 0.411
Surprise	−1.251, *p* = 0.211	−0.688, *p* = 0.492	−0.470, *p* = 0.639	−0.128, *p* = 0.898	−0.488, *p* = 0.625
Fear	−0.987, *p* = 0.324	−0.184, *p* = 0.854	**−2.567**, ***p*** **=** **0.010**	−0.688, *p* = 0.492	−0.643, *p* = 0.520
Anger	−0.978, *p* = 0.328	−0.428, *p* = 0.669	−1.126, *p* = 0.260	−0.615, *p* = 0.538	−0.630, *p* = 0.528
Disgust	**−2.043**, ***p*** **=** **0.041**	−0.825, *p* = 0.409	−0.000, *p* = 1.000	−0.197, *p* = 0.844	−1.221, *p* = 0.222
Joy	−0.209, *p* = 0.935	−0.471, *p* = 0.637	−1.279, *p* = 0.201	−0.501, *p* = 0.617	−1.351, *p* = 0.177
Sadness	−0.625, *p* = 0.532	−0.240, *p* = 0.810	−0.954, *p* = 0.340	−1.132, *p* = 0.258	−0.646, *p* = 0.518
All	−0.251, *p* = 0.802	−0.163, *p* = 0.870	−1.246, *p* = 0.213	−0.825, *p* = 0.409	−1.067, *p* = 0.286

*Significant differences in bold, being the number of correct answers higher for the front camera in both cases*.

### 3.4. Influence of Interpersonal Distance in the Evolution of the Number of Emotion Identification Errors

**Figure 6:**
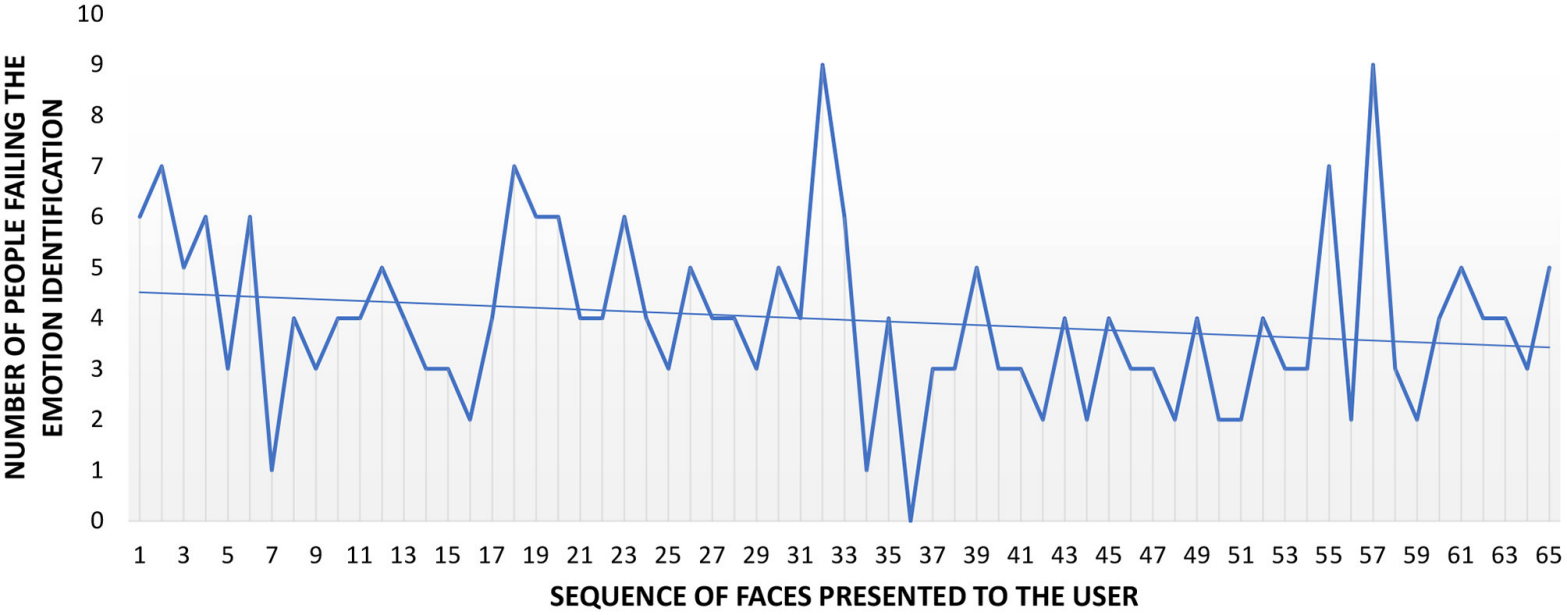
Emotion identification errors. The trend line shows a negative slope of 1.7%.

In our attempt to study whether the number of errors increased or decreases as the test progressed on each distance, we started studying the results for the combination of all of them. Later, we would analyze it for each one of them. The number of errors in emotion identification for each face presented to the participants is plotted in Figure 6, and the average value is *M* = 0.79, *SD* = 0.88. The X-axis of the graph shows the faces presented to the participants (from 1 to 65), while the Y-axis represents the number of people failing in the identification of the emotion presented. It is worth noting that the order of presentation of the emotion was different for each participant. Therefore, two situations are possible, participants made more errors at the beginning of the test (thus, they learned during the execution and improved in emotion identification) or participants made more errors at the end (they became tired). In Figure 6, the trend line shows a reduction in the number of errors as the test progressed. The slope of the line is −0.017, which is a reduction of 1.7% in the number of errors. Therefore, there is a reduction in the number of errors, but barely noticeable.

**Figure 7:**
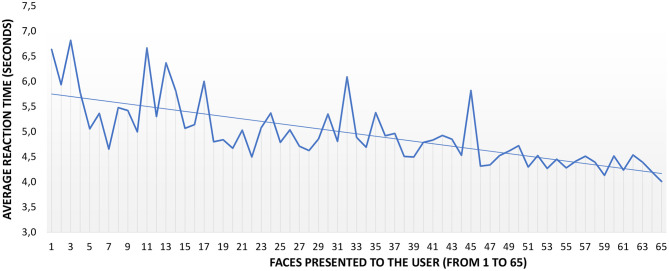
Average reaction time for each face presented to the users. The trend line shows a negative slope of 2.5%.

Regarding the influence of the IPD to the stimuli, the average number of errors per distance and face presented was *M* = 0.74, *SD* = 0.85 for D1, *M* = 0.86, *SD* = 0.79 for D2, *M* = 0.66, *SD* = 0.89 for D3, *M* = 0.82, *SD* = 0.81 for D4, and *M* = 0.91, *SD* = 1.04 for D5. This information is plotted in Figure 7, where only the trend lines are visible for the sake of clarity. These trend lines are almost straight (slope close to 0) for distances D1 to D4, but it is slightly bigger for D5 (−1.4%). However, there is no difference in the number of identification errors per distance [Friedman $\chi_{\left(4\right)}^{2}=4.770,p=0.312$].

### 3.5. Influence of Interpersonal Distance in the Reaction Time

**Figure 8:**
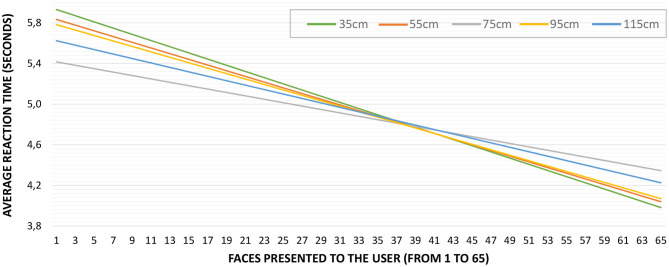
Emotion identification errors per distance. Only trend lines are displayed for clarity. The trend lines show a negative slope between −3.5% for D1 (35 cm) and −1.6% for D3 (75 cm).

Similarly to the number of errors, the influence of distance in the reaction time of the participants was also studied, and started by obtaining the average reaction time for each face presented to the participants. The average reaction time was *M* = 4.92*s, SD* = 1.03. Figure 8 shows the average reaction time of the participants as the experiment progressed. The figure plots in the X-axis the faces to be identified (from 1 to 65) by the participants, while the Y-axis shows the average reaction time. Notice that, again, the order of presentation of emotions differed from one participant to another. The slope of the trend line is −0.0247, which means a slight reduction in the reaction time during the progression of the test (2.5%).

**Figure 9:**
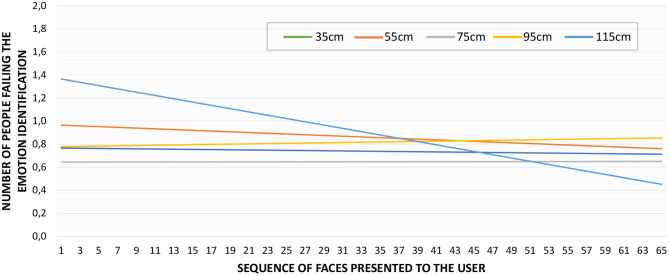
Average reaction time for each face presented to the users. Only trend lines are displayed for clarity.

Table 7 summarizes the average reaction times per IPD, which are all similar, *M* = 4.96*s, SD* = 1.08 for D1, *M* = 4.94*s, SD* = 1.23 for D2, *M* = 4.88*s, SD* = 0.79 for D3, *M* = 4.92*s, SD* = 1.07 for D4, and *M* = 4.92*s, SD* = 0.96 for D5. As discovered through these values, no significant differences in reaction time per distance was found [Friedman $\chi_{\left(4\right)}^{2}=1.059,p=0.901$]. The graph in Figure 9 presents the average reaction time of the participants as the test progressed (per each face in the sequence from 1 to 65 regardless of the presentation order). For clarity, this figure shows only the trend lines, which are all negative. Still, a small reduction in the reaction time is noticeable, −3.5% for D1, −2.8% for D2, −1.6% for D3, −2.7% for D4, and −2.2% for D5.

**Table 7:** Results for the average reaction time and standard deviation per IPD. The average reaction time for all the IPDs is *M* = 4.92*s, SD* = 1.03.

**D1**	**D2**	**D3**	**D4**	**D5**
4.96 s (1.08)	4.94 s (1.23)	4.88 s (0.79)	4.92 s (1.07)	4.92 s (0.96)

### 3.6. Influence of Interpersonal Distance in Emotion Identification Rates and Reaction Time

A partial correlation was run to determine the relationship between emotion identification rates and reaction time whilst controlling for interpersonal distance. Zero-order correlations showed that there was a statistically significant, moderate, negative correlation between emotion identification rates and reaction time [*r*(192) = −0.216, *N* = 192, *p* = 0.003], indicating that interpersonal distance had very little influence in controlling for the relationship between emotion identification rates and reaction time.

## 4. Discussion

The results of the experiment reported a high success rate in overall emotional identification (90.33%), especially for *Neutral* expression and *Joy* and *Anger* emotions, exceeding the hit rate of a previous study published by our research group in which the same stimuli were administered in a non-immersive setup (García et al., 2020b). The results show that IVR does not interfere with the emotional recognition task. In fact, IVR seems to be a more ecological instrument, which can be used as a training tool for emotional recognition. Compared to other research teams that have investigated emotional recognition using virtual humans in VR (both non-immersive and immersive), our results yield better emotional recognition rates (Dyck et al., 2008; Krumhuber et al., 2012; Gutiérrez-Maldonado et al., 2014; Amini et al., 2015; Faita et al., 2016).

With respect to the general objective of the study to identify the ideal IPD between participant and avatar in IVR settings, it can be observed that the overall emotional recognition rates for all selected distances (D1–D5) are very similar, ranging from 88.83 to 92.12%. Therefore, we cannot conclude which IPD is better in a statistically significant manner. Despite this, there is a trend that enables us to propose D3 (75 cm) as a good starting point. This is the IPD with the best overall emotional recognition rate, especially for the negative emotions *Anger* and *Sadness*. For the two positive emotions included (*Joy* and *Surprise*), D1 has the highest recognition rate. This slight trend coincides with the results obtained in previous studies which indicated that for the facial expression of a negative emotion such as *Anger*, the IPD increases, while the IPD is lower for positive emotions (Hall, 1963; Marsh et al., 2005; Welsch et al., 2020). As summarized in a very recent paper by Coello and Cartaud (2021), identifying emotional facial expressions helps to determine whether others have positive intentions or may represent a potential threat. In addition, it was revealed that positive facial expressions foster approach behaviors, whereas negative facial expressions lead to avoidance and withdrawal, resulting in a decrease or increase in IPD, respectively.

After grouping the emotions into affect categories, and subsequently studying the two positive emotions separately, it was observed that the totally extraneous behavior at intermediate distances was due exclusively to *Surprise*. This might be due to the fact that this particular emotion has been depicted as a pre-affective state or as an emotion that can be both positive and negative (Noordewier and Breugelmans, 2013). For this reason, the results on *Surprise* should be taken with care. Focusing on the purely positive emotion *Joy*, there was a clear tendency for the number of identified emotions to decrease as the IPD between avatar and participant increased, at least from D1 to D4, followed by a flattening from D4 to D5. Something similar, but in a completely opposite way, occurred with negative emotions. There was a tendency for the number of correct ratings to increase as the IPD increased from D1 to D3. There was a slight decrease (stagnation) from distance D3 to D4. This leads us to emphasize once more that positive emotions are better perceived at short distances, whereas negative emotions are better perceived at more distant distances. In the case of neutral expression, a uniform identification rate was observed from D1 to D4, followed by a small decrease. All this evidences that D3 (75 cm) would seem to be a candidate to ideal IPD offering excellent results for both positive and negative emotions, as well as for neutral expression. In order to use the set of avatars for remediation of deficits in social cognition, this IPD should be chosen to compare identification rates between healthy individuals and patients.

As in our previous study with non-immersive VR (García et al., 2020a), a higher hit rate was found for the frontal view in comparison to the lateral view. The frontal orientation provides more information about facial features, which favors successful emotional recognition. After analyzing the influence of distance and camera angles on emotion identification, we found no significant differences in either general or emotion-specific emotional recognition in the different IPDs presented. As the experiment progressed for the participants, a very slight reduction in the number of errors was observed, as well as in reaction times, with no significant differences found between the different IPDs. This suggests a certain learning effect. Moreover, this negative correlation between accuracy and response time could indicate that participants take less time to answer and guess more accurately as they learn. In addition, this learning effect is promising for the design of future psychotherapeutic treatments for the remediation of facial affect recognition deficits.

The present study has some limitations. It has not analyzed differences in terms of gender, age or educational level for the two reasons explained below. Firstly, in our previous study using a sample of 204 healthy participants we found no significant differences in this regard. Secondly, the sample included in this study does not allow for an in-depth analysis of these factors. Other important issues to be addressed regarding some weaknesses of this study are extending the range of emotions to nonbasic ones and evaluating the proposal with more sets of avatars in IVR. Regarding the maximum IPD (115 cm) used in this study, as the characteristics of HMD optical and display technologies are evolving rapidly, it is hoped that this distance restriction will be overcome in the near future. Finally, the results obtained are specific to the set-up used (combination of avatars, emotions, and HMD).

## 5. Conclusions

Considering the results presented in this study, we can conclude, in first place, that IVR allows us to reliably assess facial emotion recognition using dynamic avatars. IVR environments are more valid and ecologically friendly compared to the use of other facial stimuli used so far. However, we were not able to identify an ideal IPD; as there were no significant differences between distances on emotion recognition overall, with this particular data set, with a headset with these parameters, implying that any of these distances could be effective. Despite that, based on trends with secondary data analysis, we propose an IPD of 75 cm as a good starting point, although given the number of tests performed, these findings need to be validated by other works. This could revolutionize emotional identification approaches in experimental settings in the coming years. Secondly, knowing the ideal IPD will increase the knowledge about facial emotion recognition and will clear up some of the remaining unanswered questions. Previous studies with virtual humans in healthy population have used different avatar-participant IPDs, which could have influenced the emotion recognition rate obtained. In turn, replicating this same study in a population with different mental disorders would allow us to design useful, well-tolerated and participant-adapted assessment and intervention strategies for facial emotion recognition.

## Data Availability Statement

The raw data supporting the conclusions of this article will be made available by the authors, without undue reservation.

## Ethics Statement

The studies involving human participants were reviewed and approved by Committee on human experimentation of Complejo Hospitalario Universitario de Albacete. The patients/participants provided their written informed consent to participate in this study.

## Author Contributions

PF-S and AF-C designed the experiment. JdA, MJ, and LG-G implemented the experiment. PF-S and AG analyzed the data. JdA, PF-S, AF-C, and AG wrote the manuscript. All authors contributed to the article and approved the submitted version.

## Conflict of Interest

The authors declare that the research was conducted in the absence of any commercial or financial relationships that could be construed as a potential conflict of interest.
